# Vacuum assisted closure for the treatment of complex wounds and enterocutaneous fistulas in full term and premature neonates: a case report

**DOI:** 10.1186/s13052-016-0210-6

**Published:** 2016-01-11

**Authors:** Filomena Valentina Paradiso, Lorenzo Nanni, Laura Merli, Erika Adalgisa De Marco, Vincenzo Davide Catania, Alessandra Taddei, Carlo Manzoni, Giorgio Conti

**Affiliations:** Division of Pediatric Surgery - Catholic University of the Sacred Heart School of Medicine, Largo Agostino Gemelli, 8, 00168 Rome, Italy; Pediatric Intensive Care Unit - Catholic University of the Sacred Heart School of Medicine, Largo Agostino Gemelli, 8, 00168 Rome, Italy

**Keywords:** Preterm, Dehiscence, VAC therapy, Enterocutaneous fistula, Plastic surgery, Pediatric surgery

## Abstract

**Background:**

The Vacuum Assisted Closure (VAC) system has become an effective treatment for acute and chronic wound defects. Although its use has been reported in wound care of children and premature infants, the management of the device in this population has not been well established.

**Case presentation:**

We report the satisfactory results in two neonates (one full-term and one preterm) with complex wounds secondary to major abdominal surgery. In the premature baby an enterocutaneous fistula was also present. Complete epithelialization of the wounds was achieved in both patients within a few weeks thus avoiding any further surgical procedure.

**Case presentation:**

The use of VAC system in neonates is safe and effective in the management of complex wounds and should be considered as a first line treatment in the event of a major dehiscence.

## Background

After the initial description by Morykwas et al. in 1997 [1, 2], VAC system has become widely accepted for the cure of complex wounds. Its effectiveness in accelerating wound healing has made its use largely diffuse in the adult population. Nowadays the application of VAC in infants and newborns is progressively increasing [3–12] and indications for its use are expanding [9–13]. VAC promotes wound healing by removing localized edema, (which improves vascular and lymphatic flow), by reducing bacterial density, by enhancing angiogenesis and by increasing granulation tissue formation.

### Case presentation: Case 1

**Fig. 1:**
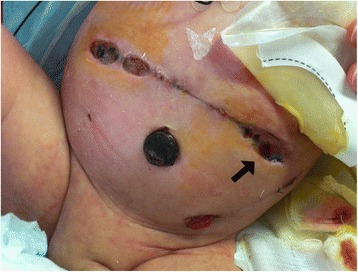
Three disctinct dehiscences are evident along the surgical wound; the arrow indicates the one in which the defect entails all the layers of the abdominal wall

**Fig. 2:**
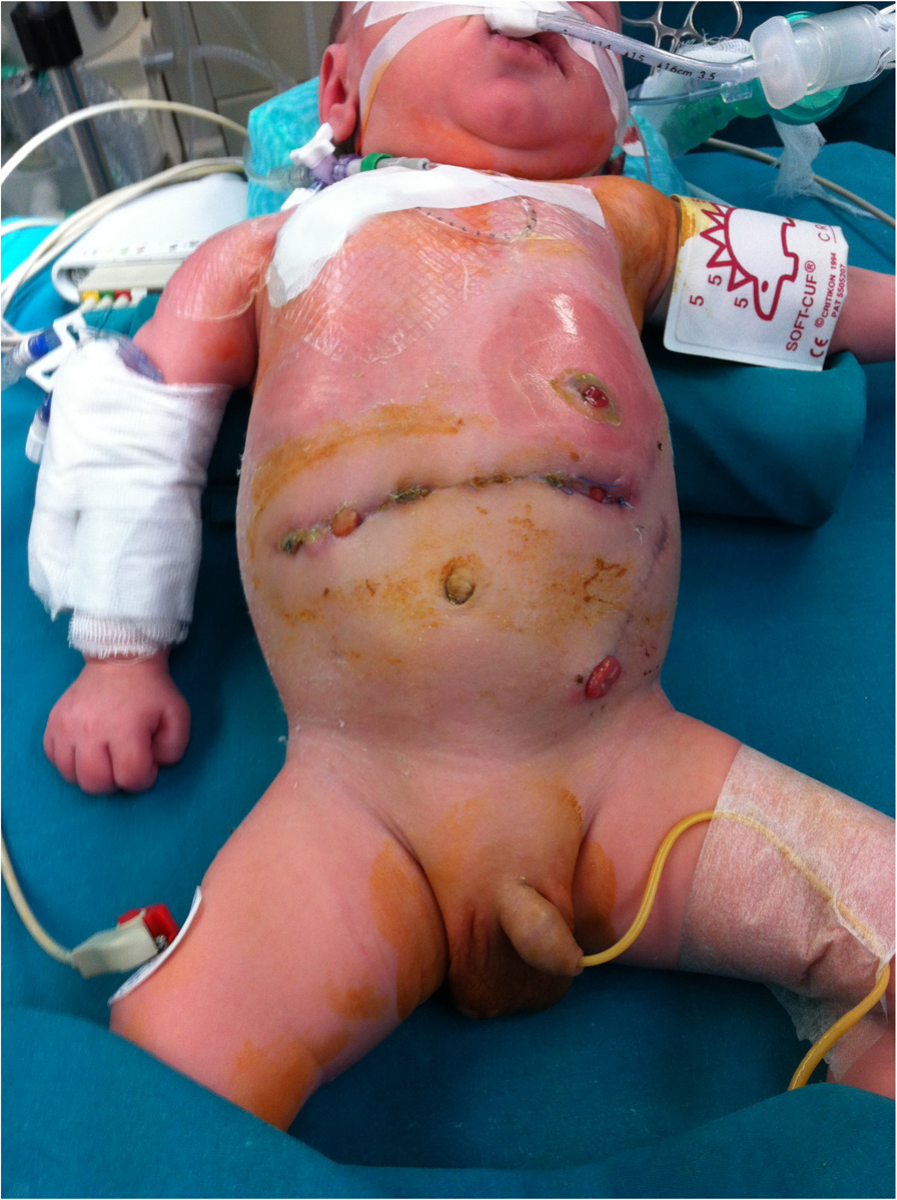
Wound healing after 19 days of treatment. Two small granulomas are evident along the scar

A full term boy (36 weeks gestational age, birth weight 2290 g) was born with ano-rectal malformation. On day of life 2 a sigmoid colostomy with separated stomas was performed in the left inferior quadrant. On postoperative day 2 the baby developed signs of bowel obstruction. At laparotomy a midgut volvulus was found and after derotation a loop jejunostomy was performed in the left upper quadrant. Total parenteral nutrition was started. In the following hours a severe metabolic acidosis ensued requiring surgical re-exploration. Due to bowel necrosis a 30 cm segment of ileum was resected and a double-barrel jejunostomy was performed at the site of the previous jejunostomy. The patient required ventilatory support for four days postoperatively. On post-operative day 7 the baby developed dehiscences (about 3 cm each) in three different sites of the surgical wound; in the left lateral side the dehiscence involved all the layers of the abdominal wall and an intestinal loop was visible protruding through the defect **[**Fig. 1**]**. VAC dressing was applied to the surgical wound including the three dehiscences. VAC GranuFoam (R) Dressing (KCI USA, Inc. San Antonio, TX 78219 USA) was modeled on the three defects and covered by a large layer of VAC drape; suction was set at −75 mmHg. In the left lateral part of the wound, where the intestinal loop was exposed, a small sheet of VAC drape was interposed to the GranuFoam in order to avoid applying suction directly to the bowel. The dressing was changed at 48 h intervals for 12 days after which the fascial defect was healed and the other defects almost completely resolved. After VAC dismission Aquacel (R) Foam Dressing (ConvaTec 100 Headquarters Park Dr Skillman New Jersey 08558 USA) was applied to the wound for 7 days until it was completely epithelialized **[**Fig. 2].

### Case 2

**Fig. 3:**
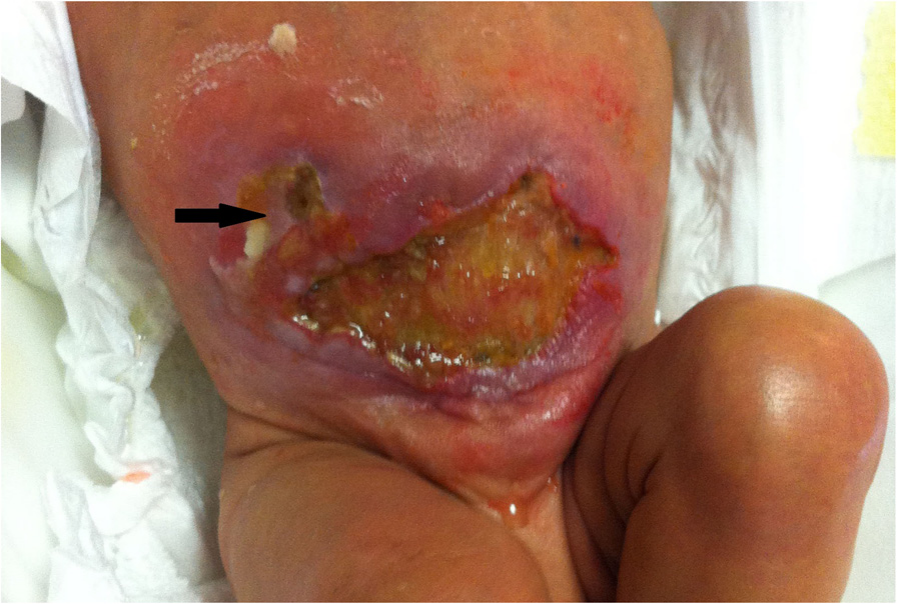
The dehiscence involves the lower abdomen almost entirely. The defect extends to the fascia which appears intact. The arrow indicates the enteric fistula in the right flank

**Fig. 4:**
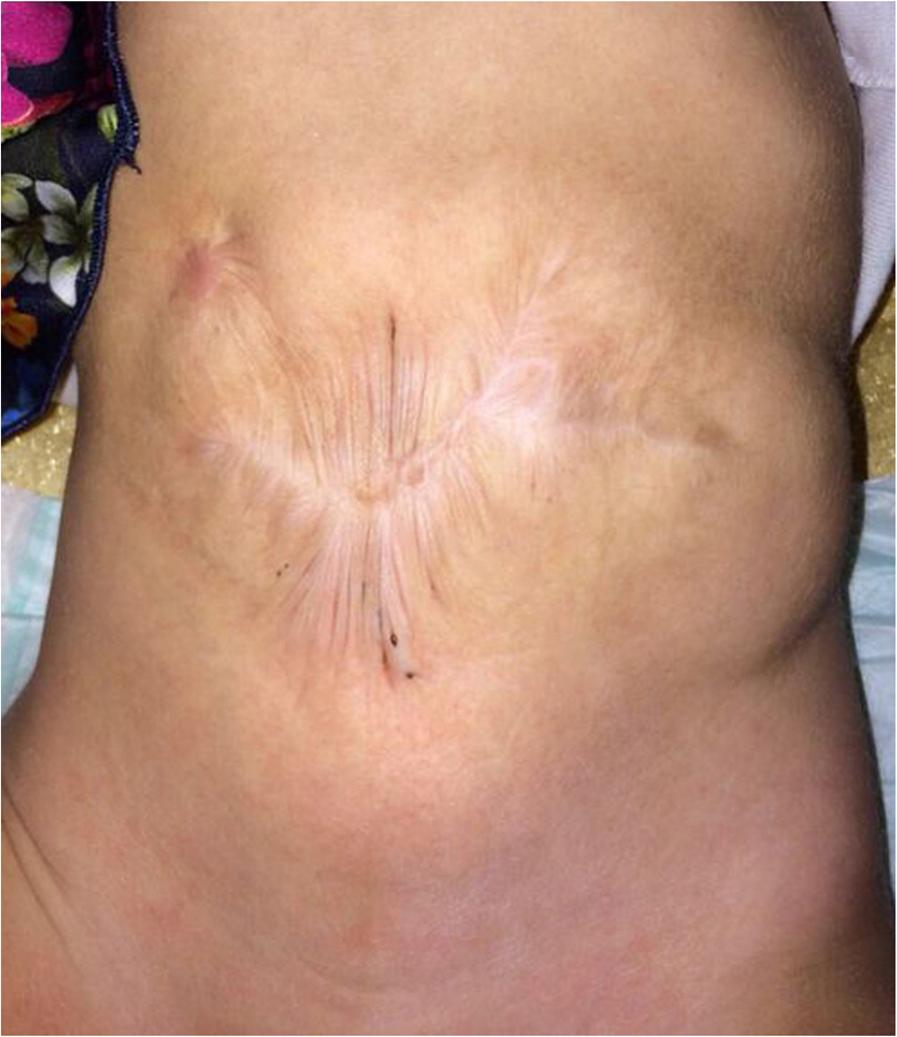
Same baby at one year follow up. The fistula has completely closed; a left incisional hernia is evident

A 24-weeks gestational age, (birth weight 650 g) female newborn underwent laparotomy for intestinal perforation at 7 days of life. Multiple perforations were found involving a 30 cm segment of ileum proximal to an ileal atresia which had not been suspected prenatally. Ileal resection and double barrel ileostomy were performed. Postoperatively the patient required ventilatory support, fluid resuscitation, and inotropic therapy. Ileostomy was taken down after three months. A few days after surgery a wide dehiscence of the surgical wound developed extending for 40 % of the abdominal wall surface **[**Fig. 3**]**. On the 16th postoperative day a low-output enterocutaneous fistula appeared in the right upper quadrant. Initially the wound and the fistula were managed with daily wet-to-dry dressing changes but no improvement was observed in the healing process of both. In addition fluid loss through the wound and the fistula was difficult to quantify and the viable skin around the defect was progressively macerating up to an 8x5 cm defect. One month after surgery a VAC dressing was applied. The VAC GranuFoam was modeled over the abdominal wall defect and suction was set at – 50 mmHg. A piece of VAC drape was interposed between the fistula and the foam in order to avoid applying suction directly to the fistula. The dressing was initially changed every 3 days for 20 days. Due to the steadily high fluid loss from the fistula and the wound the suction was then increased to −75 mmHg and the dressing was changed every 48 h for the next 21 days. When a significant drop in fluid losses was observed the VAC dressing was dismissed and Aquacel was applied for further 12 days until the fistula healed completely and the wound was completely epithelialized. At 1 year follow up the baby shows a left incisional hernia and the enterocutaneuos fistula is closed **[**Fig. 4**]**.

## Conclusions

The Vacuum assisted closure (VAC) is a widely accepted device for the treatment of chronic and acute wounds in adult population; its use is progressively extending to the pediatric patients for the treatment of dehiscences and a wide spectrum of different pathologies.

In 2000 Moneey et al. [7] reported 27 pediatrics patients (age range 3 days to 18 years) in which the VAC system was used for the treatment of soft tissue defects resulting from acute or chronic extremity or axial wounds that failed primary surgical closure. The healing was obtained either by the exclusive use of the device or by adding a skin graft or flap after an adequate granulation tissue had developed. The Authors underlined the advantage of obtaining a wide surface of granulation tissue in a short time and the possibility to avoid extensive microvascular tissue transfer.

Similar favorable results were reported by Butter et al. [8] who described 16 children (range 1 month-18 years) receiving VAC treatment. Indications in this population were not only wound dehiscences (abdomen, sternum, back and leg) but also extensive tissue loss after pilonidal sinus excision, and chronic postoperative perineal fistulas. Wound closure occurred in 15 out of 16 patients and mean healing time was 28 days in case of dehiscences. Time to achieve complete healing was longer in recurrent pilonidal sinuses compared to primary excisions. In their report the Authors underline the benefits of fewer dressing changes, the faster return to daily activities and the cost-effectiveness of VAC treatment compared to daily dressings despite the higher cost of the device.

The effectiveness of VAC in pediatrics was also demonstrated by Caniano et al. [5], in 2005, who reported an optimal success rate in the management of 51 pediatric patients with different pathologies. Despite the retrospective basis of their analysis the Authors infer a better cost-effectiveness of the device compared to traditional dressings due to the documented faster healing. Seven patients out of 51 presented an extensive tissue loss of the abdominal wall and two of them were neonates. In these latter patients the healing occurred over an average period of 1 month.

The management of complex wounds in newborns is more challenging than in children. In fact such lesions can be a life-threatening problem for these patients because of the significant amount of extracellular fluid loss that can be expected due to the high surface-area-to-volume ratios. In his paper Arca [4] underscores the disadvantages related to heat loss (due to prolonged contact with the extracellular fluids) and to the fasting necessary to administer anesthesia in cases of repeated return to the operating room. All of these drawbacks are eliminated by the use of VAC system. In both of our cases no return to the operating room was necessary: all dressing changes were performed at the bedside and no analgesia or sedation proved necessary during the procedures. Additionally an accurate measurement of the fluid losses allowed appropriate restoration thus avoiding fluid and electrolytes imbalance.

The application of the VAC system to neonates implies some necessary adaptations in terms of choice of the most appropriate kind of foam, pressure applied and protection of the surrounding skin and underlying viscera.

VAC system is provided with 2 types of sponges with different cell sizes (VAC GranuFoam Dressing [black-colored sponge] or VAC WhiteFoam Dressing [white-colored sponge]; KCI, San Antonio, TX); the latter comes pre-soaked in saline and has the smallest cell size, therefore it and is thought to be less adherent than the other thus preventing excessive risk of fistula formation. In our cases the GranuFoam (largest cell size) was used but direct contact to the fascial defect and the enteric fistula was prevented by interposition of a small piece of the same adhesive drape sealing the VAC system.

Another relevant issue for application of VAC to neonates is the correct setting of negative pressure. The device can develop a negative pressure range of −25 to −125 mmHg. Though it is commonly set at −125 mmHg when used in adult patients, the recommended vacuum level for neonates is −50 to −75 mmHg. In our cases the vacuum was kept at - 50 mmHg at the beginning of VAC treatment due to exposure of an intestinal loop or the presence of an enteric fistula. Subsequently, once coverage was achieved with a thin layer of granulation tissue we modified the pressure setting to - 75 mmHg.

Some Authors [6] suggest to interpose a thin Duoderm dressing (ConvaTec, Princeton, NJ) between the skin and the plastic drape in order to protect the skin surrounding the defect from maceration or mechanical injury at removal of the plastic drape. We didn’t use this protection layer in both our cases and no skin damage was observed in any of the patients.

In order to prevent the underlying structures from being damaged by excessive vacuum a non-adherent dressing (Adaptec, Johnson and Johnson, Langhorne, PA) may be applied to the wound bed, especially in those cases in which it is difficult to determine which structures are exposed [3, 6]. In our experience protection of the enteric fistula and the exposed bowel loop was obtained by interposing a piece of the VAC drape to the modeled foam; in this way complete healing of the wound and no residual ventral hernia was obtained.

Enteric fistulas have been considered as complications of VAC system [3]. The Authors suggest to increase the vacuum in the presence of an enteric fistula providing a different collection bag for the enteric effluent. Our experience indicates that in cases of low output fistulas keeping the vacuum to a low level may help in obtaining closure of the fistula. This may be due either to the size or the output of the fistula; we think that an appropriately low negative pressure applied to the device could have played a role in the favorable result.

The optimal results in the two cases described confirm that VAC system is a safe and effective device in the treatment of complex wounds even in neonates or preterm babies.

Written informed consent was obtained from the parents for publication of this case report and any accompanying images. A copy of the written consent is available for review by the Editor-in-Chief of this journal.
